# On Strain-Hardening Behavior and Ductility of Laser Powder Bed-Fused Ti6Al4V Alloy Heat-Treated above and below the β-Transus

**DOI:** 10.3390/ma17143401

**Published:** 2024-07-09

**Authors:** Emanuela Cerri, Emanuele Ghio

**Affiliations:** Department of Engineering for Industrial Systems and Technologies, University of Parma, Via G. Usberti, 181/A, 43124 Parma, Italy; emanuela.cerri@unipr.it

**Keywords:** work-hardening rate, strain-hardening, titanium alloy, laser powder bed fusion, mechanical properties, uniform elongation, work-hardening capacity

## Abstract

Laser powder bed-fused Ti6Al4V alloy has numerous applications in biomedical and aerospace industries due to its high strength-to-weight ratio. The brittle α′-martensite laths confer both the highest yield and ultimate tensile strengths; however, they result in low elongation. Several post-process heat treatments must be considered to improve both the ductility behavior and the work-hardening of as-built Ti6Al4V alloy, especially for aerospace applications. The present paper aims to evaluate the work-hardening behavior and the ductility of laser powder bed-fused Ti6Al4V alloy heat-treated below (704 and 740 °C) and above (1050 °C) the β-transus temperature. Microstructural analysis was carried out using an optical microscope, while the work-hardening investigations were based on the fundamentals of mechanical metallurgy. The work-hardening rate of annealed Ti6Al4V samples is higher than that observed in the solution-heat-treated alloy. The recrystallized microstructure indeed shows higher work-hardening capacity and lower dynamic recovery. The Considère criterion demonstrates that all analyzed samples reached necking instability conditions, and uniform elongations (>7.8%) increased with heat-treatment temperatures.

## 1. Introduction

Ti6Al4V is a titanium alloy increasingly used in aerospace and biomedical applications due to its excellent combination of high strength, corrosion resistance, high fatigue life, and toughness [1,2,3,4]. Due to its high strength-to-weight ratio, Ti6Al4V is undoubtedly used to manufacture space capsule components, compressor blades, helicopter rotor hubs, and orthopedic and cranial implants. In this scenario, in which customization and flexibility are among the main design requirements, the laser powder bed fusion (LPBF) process finds a wide application area. In fact, the melt and fusion process of the metallic powder in a layer-by-layer methodology makes it possible to manufacture components with complex and topology-optimized geometry [5,6]. The LPBF process is also motivated by the absence of tools and minimal post-processing machining requirements [7]. 

Ti6Al4V is an α + β alloy where the α-stabilizers (Al, O, N, C) and β-stabilizers (V, Fe) stabilize the hcp (hexagonal close-packed) α-phase and the bcc (body-centered cubic) β-phase, respectively, at room temperature. Due to the nonequilibrium solidification process that occurs during the LPBF process, the as-built microstructure of the Ti6Al4V samples is composed of a hierarchical structure of needle-like α′-martensite laths arranged within columnar prior-β grains. In detail, β-grains nucleate directly on the build platform and grow from the bottom region to the top one, following the several solidified powder layers (i.e., towards the molten pools). The diffusionless β → α′-martensite transformation takes place within each molten pool [6,8,9,10]. Thanks to these microstructural features, laser powder bed-fused Ti6Al4V alloy shows higher strengths but lower work-hardening and ductility than wrought Ti6Al4V alloys, thus limiting its applications [6,11,12]. At the same time, the fast and localized cycles of heating and cooling caused by the laser–powder interactions trigger differential expansion and contraction of localized zones of the manufactured component. This generates stresses and strains that remain within the components as residual stresses and strains [6,13]. 

Post-processing heat treatments can improve the ductility of the as-built Ti6Al4V due to the following reasons [3,6]:

1. Decomposition of the brittle α′-martensite laths into α + β phase during the exposure at temperatures below the β-transus, i.e., during stress relief, annealing, and sub-transus solution heat treatment (SHT). Ductility improves with increasing heat-treatment temperatures.
2. Recrystallization of the columnar β-grains during the exposure at solution temperatures (above the β-transus) and the formation of a desired α + β microstructure by controlling the cooling pathway from the β-region to the room temperature.

For example, Vracken et al. [14] showed that the strain of as-built LPBFed Ti6Al4V alloy (e = 7 ± 1%) increases by 25% after annealing at 705 °C per 3 h, and by 85% in slowly cooled samples (furnace) from 1020 °C. When the solution-heat-treatment temperature increases up to 1150–1200 °C, no ductility enhancement is observed, as summarized in [6]. 

Focusing on the mechanical strength, both the as-built YS (YS, 980 ÷ 1200 MPa) and UTS (UTS, 1100 ÷ 1300 MPa) decrease with an increase in the heat-treatment temperature. For the solution-heat-treated samples, both UTS and YS can be recovered due to the β → α′-martensite transformation that occurs during a rapid cooling [15]. 

Work-hardening (i.e., strain-hardening) is closely related to the characterization of the plastic deformation of metallic materials, as well as strength, deformability, toughness, and ductility [16,17,18]. It is fundamentally based on an intricate interaction between several microstructural features such as grain boundaries, misorientation, dislocation, and crystal lattice [19,20]. In cubic structures, the strain-hardening behavior is well understood, with the main hardening mechanism primarily based on the accumulation of a dislocation forest. On the contrary, the significant plastic anisotropy and the low symmetry characterizing the hcp (hexagonal close-packed) lattice complicate the characterization of the strain-hardening behavior [21,22]. For these reasons, the various microstructural features characterizing the Ti6Al4V alloy and the different lattice structures of both hcp α-phase and bcc (body-centered cubic) β-phase make the work-hardening analysis meaningful. Several studies have investigated the work-hardening behavior of cast Ti6Al4V at different strain rates and high testing temperatures [23,24,25,26]. In recent years, few studies have focused on the work-hardening analysis of additively manufactured Ti6Al4V alloys [7,16,27,28,29]. De Formanoir et al. [29] investigated electron beam powder bed-fused Ti6Al4V in annealed (850 °C), sub-transus SHTed (920 °C), and hot-isostatic pressing conditions. Jankowski [28] briefly analyzed the mechanical behavior of additively manufactured Ti6Al4V alloys, considering only the morphology of the work-hardening curves and the softening coefficient. Muiruri et al. [27] investigated direct metal laser-sintered Ti6Al4V after a cycle of different heat treatments. Lastly, Song et al. [7] briefly analyzed the work-hardening behavior of an LPBFed Ti6Al4V alloy in as-built conditions by comparing it to Ti44 and Ti84 alloys. 

To improve the literature review and considering the importance of the work-hardening behavior in various aerospace applications, the present study aims to evaluate the work-hardening behavior of a Ti6Al4V alloy laser powder bed-fused in different orientations and heat-treated below and above the β-transus temperature. Specially, work-hardening exponent, plastic instability, and work-hardening capacity are investigated, taking into account the microstructural variations observed after:

1. Annealing heat treatments at 704 and 740 °C;
2. Super-transus SHT at 1050 °C.

The results presented and discussed in this study build upon the microstructural investigations previously performed by the authors in [30,31]. 

## 2. Materials and Methods

Gas-atomized Ti6Al4V powder, whose chemical composition is listed in Table 1, was used to additively manufacture dog-bone test samples.

**Figure 1:**
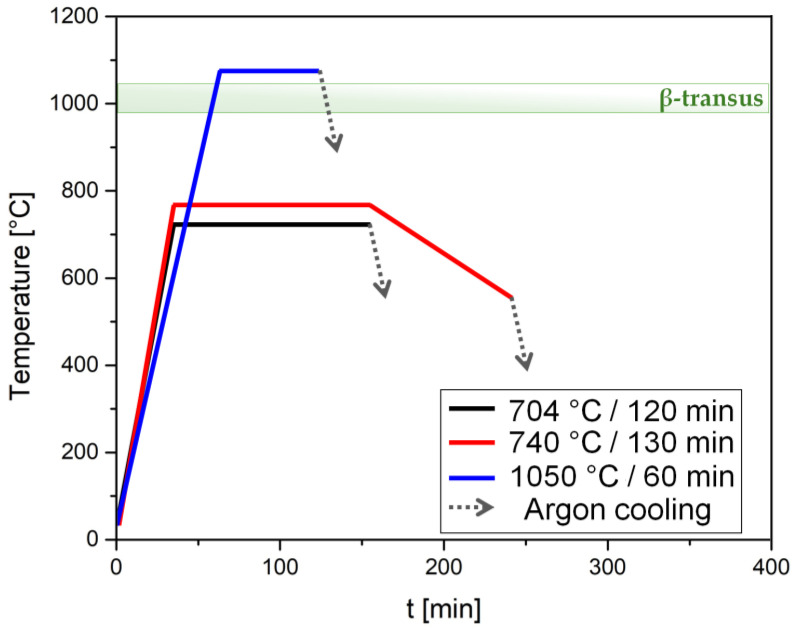
Temperature–time curves of the heat treatments performed at 704 °C × 120 min (black line), 740 °C × 130 min (red line), and 1050 °C × 60 min (blue line).

Tensile samples were laser powder bed-fused using an SLM^®^280 machine (SLM: selective laser melting, SLM Solution, Lübeck, Germany) with different orientations relative to the build platform (i.e., 0, 45, and 90 °C), utilizing the process parameters reported in [30,31]. Before their removal from the build platform, the samples were heat-treated in a vacuum furnace at temperatures below (704 °C, 740 °C) and above (1050 °C) the β-transus (Figure 1) to prevent the possible formation of cracks considering the brittleness of the as-built microstructure. As shown in Figure 1, samples heat-treated at 704 °C and 1050 °C were directly cooled in argon gas (dotted lines) for 60 min. Samples exposed to 740 °C were first furnace-cooled down to 520 °C over 90 min and then cooled with argon gas to room temperature. The annealing heat treatment at 704 °C was carried out in accordance with ASM 2801b standard [32], while that performed at 740 °C was developed to further enhance the anisotropic mechanical behavior of the as-built Ti64 alloy.

Microhardness was measured using a Vickers tester machine (VMHT Leica, Wetzlar, Germany) with a load of 100 gf and a dwell time of 15 s. The microhardness of heat-treated samples was obtained as the average of 9 indentations arranged in a 3 × 3 matrix, as discussed in [30]. 

The microstructure and fracture profiles of the tensile samples were observed through an inverted microscope (DMi8 Leica, Wetzlar, Germany). The investigated surfaces were grinded (P80-P4000), polished with silica colloidal suspension, and then chemically etched with Keller’s reagent. In-depth microstructural analysis was previously performed and discussed in our earlier works [30,31]. 

**Figure 2:**
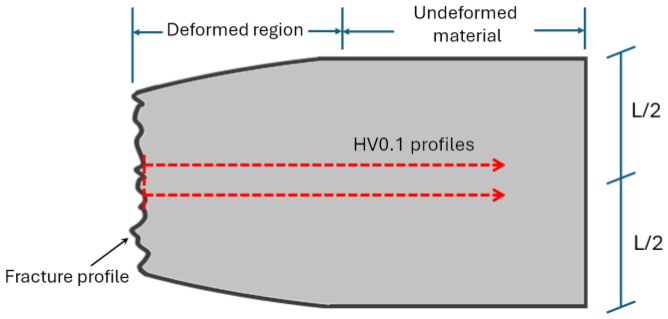
Graphical representation of the microhardness profiles performed on the cross-section of the tested samples. L represents the diameter of the dog-bone samples (L = 6.0 ± 0.1 mm).

Dog-bone samples, with a gauge length of 30 mm and a cross-sectional area of 36 mm^2^, were tensile tested at room temperature using a Zwick Z100 machine (Zwick/Roell, Ulm, Germany) at a constant strain rate of 1.6 × 10^–3^ s^–1^. Tensile tests were repeated three times for each heat-treated condition to ensure the reliability of the results. The as-built mechanical properties were obtained by the literature review. To characterize the cross-section of each tested sample, two parallel hardness profiles (red dotted lines in Figure 2) were performed. These profiles extended from the fracture surface to the zone where both profiles converged to the microhardness of the undeformed sample. The microhardness profiles were spaced at 100 µm apart.

To investigate the work-hardening behavior of the heat-treated Ti6Al4V alloy, the Ludwik–Hollomon equation was used and applied to the plastic flow region. This equation (Equation (1)) is also known as the power-law hardening equation:

(1) $$\sigma =K\varepsilon^{n}$$

where σ is the true stress (MPa), ε is the true strain (-), K is the strength coefficient (MPa), and n is the strain-hardening exponent (-). By differentiating Equation (1) with respect to the true elongation

(2) $$\frac{d\sigma }{d\varepsilon }=n\frac{\sigma }{\varepsilon }=\theta ,$$

the obtained work-hardening rate $\left(\theta \right)$ can be used to investigate the ductile effects of the matrix. If the plastic region is well approximated by the power-law (Equation (1)), the work-hardening rate (Equation (2)) will intersect the true stress–strain curves at a point representing the true UTS $\left(\sigma_{UTS}\right)$ and the respective strain value $\left(\varepsilon_{UTS}\right)$ [33]. This point also defines the instability condition that may occur during a tensile test, namely, the onset of necking. This condition meets the following equivalence [34]:

(3) $$\varepsilon_{UTS}=\varepsilon_{u}=n$$

For these observations, the power-law equation (Equation (1)) at the UTS point can lastly be rewritten as follows:

(4) $$\sigma_{UTS}=K\varepsilon_{u}^{n}=Kn^{n}$$

Through this brief contextualization, the work-hardening rate was also used to investigate the point at which the necking occurred. Newer studies [35,36] supported these statements.

**Figure 3:**
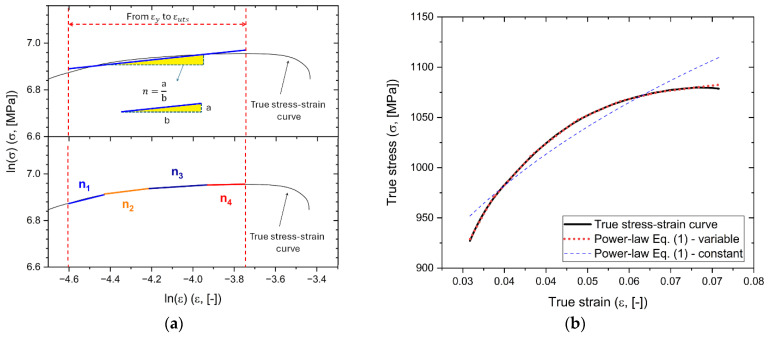
(**a**) True stress–strain curve plotted into a double-logarithmic graph to obtain the strain-hardening exponent (n) and the strength coefficient (K) as constant (first row) or variable (second row) values. (**b**) Representative true stress–strain curves, limited to the YS to the UTS, obtained by tensile test (black line), power-law (Equation (1)) where *n* and *K* are variable from YS to UTS (red dotted line), and power-law (Equation (1)) where *n* and *K* are constant.

To obtain both the strain-hardening exponent and the strength coefficient for each heat-treated condition, Equation (1) was considered in a logarithmic form:

(5) $$\ln\left(\sigma \right)=n\ln\left(\varepsilon \right)+\ln\left(K\right)$$

where n represents the slope and K is the y-intercept (i.e., when ε=1) of the linear fit (Figure 3a) carried out in the $\varepsilon_{YS}$ − $\varepsilon_{UTS}$ range. Due to the morphology of the true stress–strain curve and to better evaluate the correlation between the plastic region of the true stress–strain curve and the power-law, both the slope $\left(n\right)$ and the y-intercept $\left(K\right)$ were considered first as constant values, as shown in the single linear fit in Figure 3a (top row), and second as variable values, as highlighted by the four different linear fits in Figure 3b (bottom row). Another study [37] considered n and K as variable values due to the nonlinear morphology of the true stress–strain curve. Figure 3b shows how the true stress–strain curve (black line) can be approximated to the curves obtained by Equation (1) (i.e., power-law) so that the instability Considère criterion (Equation (2)) can be applied. For the discussions made on Equations (2)–(5), first, the true stress–strain curve is considered from YS to UTS points (namely, in the uniform elongation region). Second, the blue and red dotted lines in Figure 3b indicate the stress–strain curves obtained using the power-law equation (Equation (1)) in which the values n and K are considered first as constant (blue line in the top row of Figure 3a), and then as variable (red line in bottom row of Figure 3b). By comparing the power-law functions, the best approximation of the plastic region was evidently provided by the red dotted line, namely, when the *n* and *K* values are considered variable. 

The ratio $\left(R\right)$ between the true yield strength $\left(\sigma_{YS}\right)$ and ultimate tensile strength $\left(\sigma_{UTS}\right)$,

(6) $$R=\frac{\sigma_{YS}}{\sigma_{UTS}},$$

was used to investigate the brittleness conferred by the different microstructures. The true YS was obtained by the intersection between the true stress–strain curve and the offset line at 0.002 positive true strain from the linear portion. The UTS was obtained by applying the Considère criterion to the stress–strain curve (Equation (2)).

As previously mentioned, the Considère criterion defines the point in which the necking instability occurs and the respective UTS $\left(\sigma_{UTS}\right)$ value. To obtain the true plastic strain ($\varepsilon_{p}$ (-)) characterizing each Ti64 sample, the following integral, evaluated from $\sigma_{YS}$ to $\sigma_{UTS}$, was considered:

(7) $$\varepsilon_{p}=\int_{\sigma_{YS}}^{\sigma_{UTS}}\frac{d\varepsilon }{d\sigma }\;\cdot d\sigma$$

Observing that the derivative $\left(\frac{d\varepsilon }{d\sigma }\right)$ is the inverse of the work-hardening rate (Equation (2)), Equation (7) can be rewritten as follows:

(8) $$\varepsilon_{p}=\int_{\sigma_{YS}}^{\sigma_{UTS}}{\left[\theta \left(\sigma \right)\right]}^{-1}\cdot d\sigma$$

**Figure 4:**
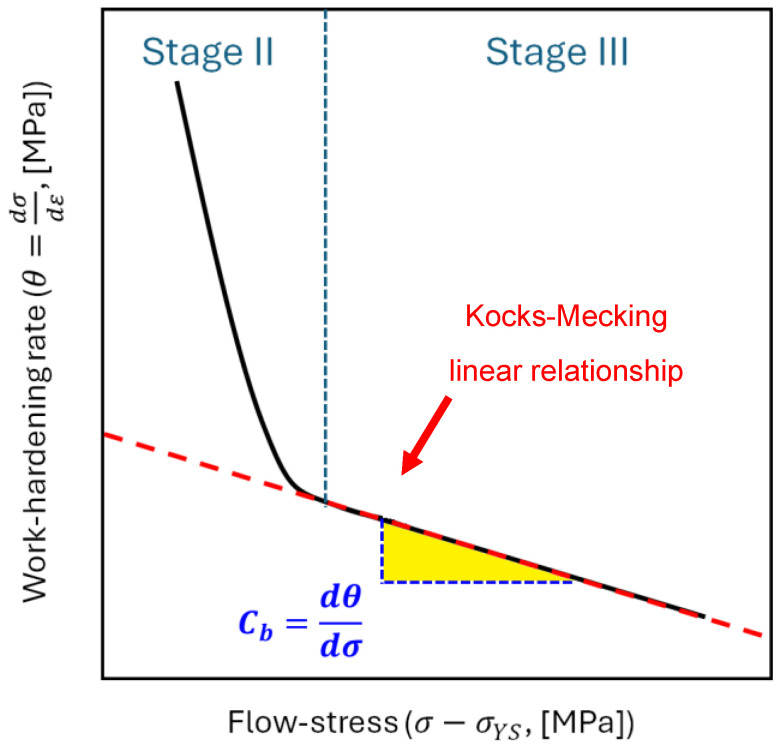
Schematic representation of the work-hardening curve (black line) elucidating the Kocks–Mecking analysis, in which the red dotted line represents the linear relationship between the work-hardening and the flow stress and Cb is its slope.

By considering and integrating the Kocks–Mecking linear relationship [22,38], Equation (8) confers the following result:

(9) $$\varepsilon_{p}=\frac{\ln\left[1+C_{b}\left(1-R\right)\right]}{C_{b}}$$

where $C_{b}$ is the softening coefficient, which is related to the dynamic recovery occurring in Stage III of the work-hardening curve (Figure 4), and R is as expressed in Equation (6). In detail, the softening coefficient $\left(C_{b}\right)$ represents the slope of the Kocks–Mecking linear relationship (red dotted line) between the work-hardening and the flow stress [22,38,39].

## 3. Results

**Figure 5:**
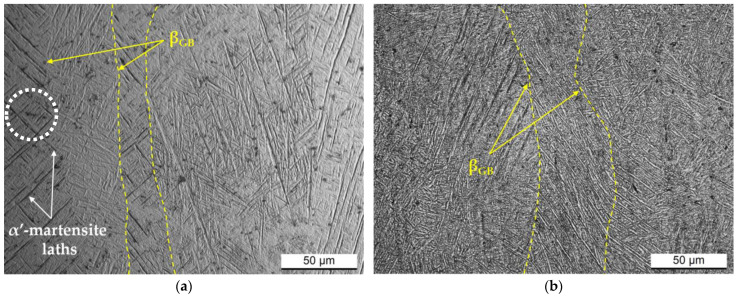
Optical micrographs acquired on the xz-plane (parallel to the build direction) of the Ti64 Z-samples in (**a**) as-built conditions and after the heat treatments at 740 °C/120 min (**b**).

Figure 5 shows the as-built (Figure 5a) and heat-treated (Figure 5b) microstructures acquired on the xz-plane of the Ti6Al4V dog-bone samples. The as-built microstructure (Figure 5a) is characterized by columnar β-grains containing hcp α′-martensite laths (white arrows in Figure 5a). As investigated in several studies [10,31,40], the LPBF process promotes, first, the nucleation of the β-grain directly on the build platform and, secondly, its growth along the build direction, namely, across the several molten pools. For this reason, columnar β-grains are disposed parallel to the build direction. The high cooling rate controlling the solidification process of the molten pools induces the diffusionless β → α′-martensite transformation and forms a cross-hatched structure of α′-martensite laths (dotted circle in Figure 5a). As deeply studied by Yang et al. [8], the cross-hatched structure is composed of a twine of several laths disposed perpendicular and/or parallel to each other. In detail, primary α′-martensite laths always extend across the whole columnar grain. Secondary, tertiary, and quartic α′-martensite laths, characterized by finer dimensions, are instead disposed of parallel and/or perpendicular to the primary ones. As widely investigated in our previous works [30,31], the heat treatments performed at temperatures below the β-transus (704 °C and 740 °C, Figure 1) induced the α′-martensite → α + β decomposition due to the diffusion of the β-stabilizer alloying elements (i.e., V, Fe) from the supersaturated hcp lattice to the α-phase grain boundary. The EBSD observations in [31] showed the presence of about 3% of the newly formed β-phase and that the newly formed α-phase retains the same orientation as its progenitor α′-martensite lath. The same EBSD maps in [31] indicated that the α-phase has an orientation relation with the retained columnar β-grain (yellow dotted line in Figure 5b). 

**Figure 6:**
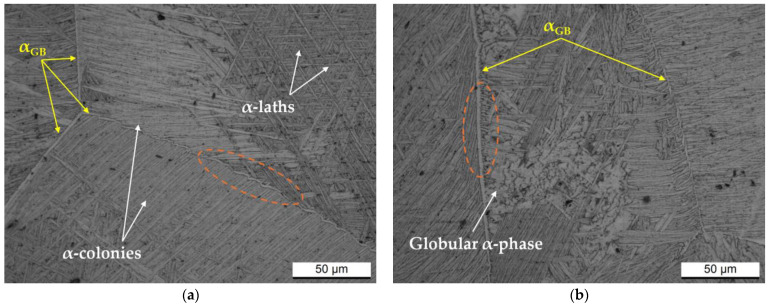
(**a**,**b**) Optical micrographs acquired on the xz-plane (parallel to the build direction) of the Ti64 Z-samples after the heat treatments at 1050 °C/60 min and representing two different zones of the same sample.

Exposure at 1050 °C (Figure 6) recrystallizes the columnar β-grains into equiaxed grains whose boundaries are formed by the α_GB_ (GB: grain boundary), thereby removing the microstructural anisotropy conferred by the LPBF process. On the other hand, the argon cooling from the β-region (Figure 1) conferred another degree of microstructural anisotropy. In fact, some newly β-equiaxed grains are formed by α + β laths distributed into a Widmanstätten structure and α + β colonies (Figure 6a), while others consist of globular or coarsened α-phase (Figure 6a). This microstructural variation is generally conferred by dissimilar cooling rates affecting the β → α + β transformation as also supported by the α_GB_ and β-phase distribution along the grain boundaries (orange dotted lines). Specially, the β-phase precipitates along the α_GB_-phase and separates it from the α + β laths or colonies (Figure 6a) during fast cooling. Conversely, lower cooling rates promote the interconnection between the α_GB_-phase and the α laths (Figure 6b). These statements are supported and well documented in [41,42].

**Figure 7:**
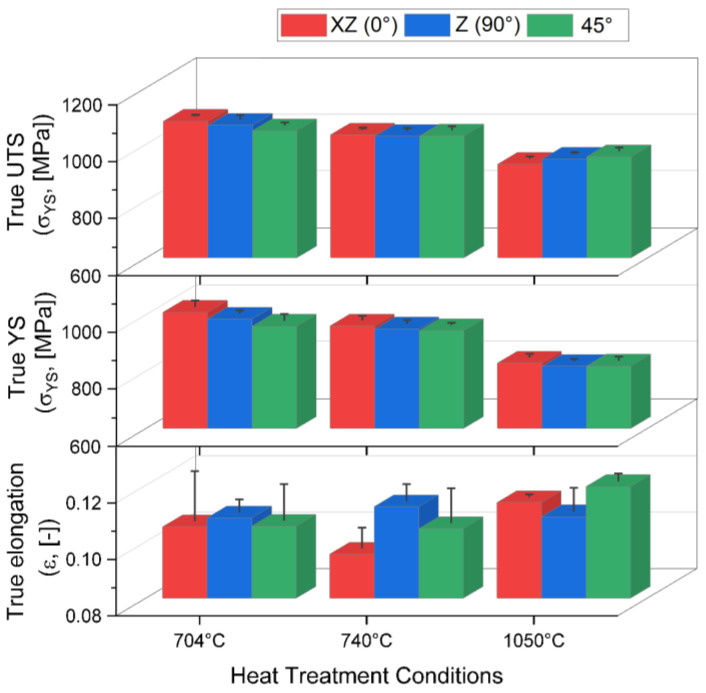
True UTS ($\sigma_{UTS}$ (MPa)), YS ($\sigma_{YS}$ (MPa)), and elongation (ε (-)) values of the Ti64 samples (XZ, Z, and 45°) heat-treated at 704 °C, 740 °C, and 1050 °C.

Figure 7 summarizes the mechanical properties trend of the XZ-, Z-, and 45-samples heat-treated at 704 °C (red columns), 740 °C (blue columns), and 1050 °C (green columns). Generally, the heat treatments at both 704 °C and 740 °C reduced the as-built UTS (σ_UTS_ > 1.1 GPa, [6,9,43,44,45]) and YS (σ_YS_ > 950 MPa, [6,9,43,44,45]) values due to the α′-martensite → α + β decomposition. Considering that the α′-martensite decomposition typically occurs above 400 °C, the furnace cooling from 740 °C to 520 °C continues to increase the amount of the decomposed martensite [6,46]. For this reason, and because of the coarsening phenomena affecting the α-phase (according to the Hall–Petch law), both the YS and UTS decrease from the samples annealed at 704 °C to those at 740 °C (Figure 7, Table 2). Our previous work [30] showed that increasing the heat-treatment temperatures from 704 to 740°C, and varying the cooling pathway, resulted in an increase in grain size from (540 ± 60) nm to (799 ± 10) nm. For the same findings, UTS and YS values slightly decreased (Figure 7, Table 2) when the heat-treatment temperature increased from 704 °C to 740 °C. The highest strength reduction and the anisotropy removal were conferred by the SHT at 1050 °C per 60 min because of the recrystallized microstructure shown in Figure 6. Contrary to the expectation, the elongation values did not exhibit an adequate increment relative to the strength reduction, likely due to the presence of:

1. The fine α + β laths and colonies;
2. The α-case layer (as will later be discussed).

Similar findings were observed in [6,47,48]. 

**Figure 8:**
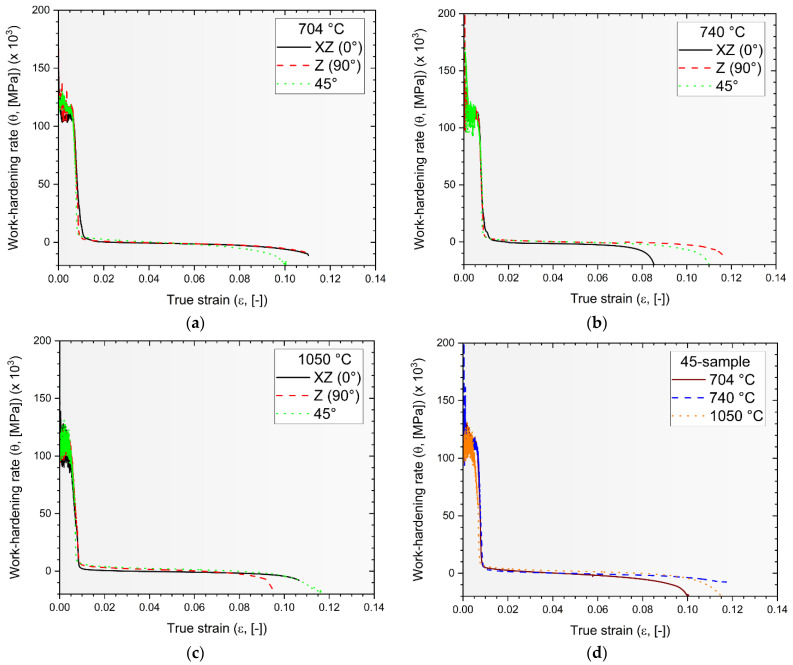
Work-hardening curves of the Ti64 samples heat-treated at 704 °C (**a**), 740 °C (**b**), and 1050 °C (**c**). The graph in (**d**) displays the work-hardening curves of the 45-sample heat-treated at 704 °C (red line), 740 °C (blue line), and 1050 °C (orange line).

Figure 8 shows the work-hardening curves obtained by considering the variable strain-hardening exponents and applying Equation (2) to the Ti6Al4V samples heat-treated at 704 °C (Figure 8a), 740 °C (Figure 8b), and 1050 °C (Figure 8c). It is important to note that the work-hardening behavior of a polycrystalline metallic material is closely related not only to the grain size and distribution, dislocations, and misorientations, but also to dislocation annihilation, formation of local shear zones, and new sub-grains. These factors influence the stages of the work-hardening. Each curve presents the same three distinct stages (Stages I–III). Stage I describes the dislocation multiplication, leading to rapid decrease in the work-hardening rate, with an increase in plastic strain [49]. Immediately after this stage, towards the end of the elasto/plastic region, Stage II shows an increase in the work-hardening rate up to a relative maximum, possibly due to the possible presence of stacking faults or twins, as investigated in [50,51,52,53]. Stage III is dominated by the dynamic recovery (represented by $C_{b}$ in Figure 4), where dislocation annihilation occurs. Finally, the sudden decrease in the work-hardening rate (Stage IV) is not well described in the literature yet. Muiruri et al. [27] suggested that intense localization of shear described the final part of the work-hardening curve. Considering the shape of the curves, it can be concluded that the microstructural variations (Figure 5) resulting from the heat treatments did not significantly affect the strain-hardening behaviour.

Despite the similarity between the work-hardening curves (Figure 8a,b vs. Figure 8c), the work-hardening rate, calculated as widely described in [54], decreases as the heat-treatment temperature increases from 704 °C to 1050 °C, as listed in Table 3. Simultaneously, the orange curve plotted in Figure 8d exhibits a slower work-hardening rate than those described by the red and black curves in the same Stage II. The smaller number of dislocations within the lattice structure of the SHTed Ti6Al4V sample may support these findings. As affirmed in [55], additively manufactured Ti6Al4V alloy heat-treated below the β-transus (<800 °C) showed a higher amount of dislocation compared to that observed in over-transus SHTed samples. Lastly, in accordance with the expectations, in samples annealed at 704 °C and 740 °C, the equality between the black and red work-hardening curves in the first stage indicates that there is no significant variation in dislocation multiplication.

**Figure 9:**
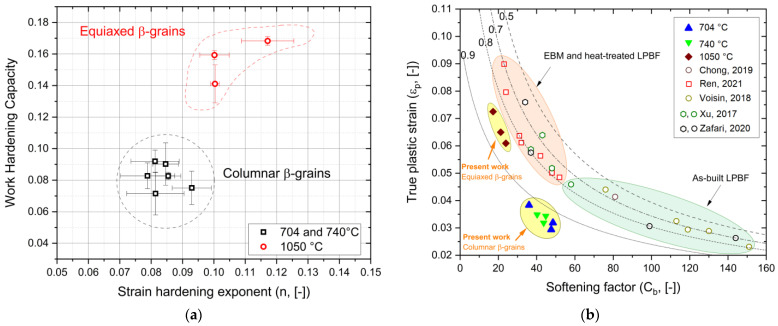
(**a**) WHC versus strain-hardening exponents of the Ti64 samples heat-treated at 704 °C, 740 °C, and 1050 °C. (**b**) Variation of the softening factor in relation to the true plastic strain of the samples analyzed in the present work and in [53,60,61,62,63].

**Figure 10:**
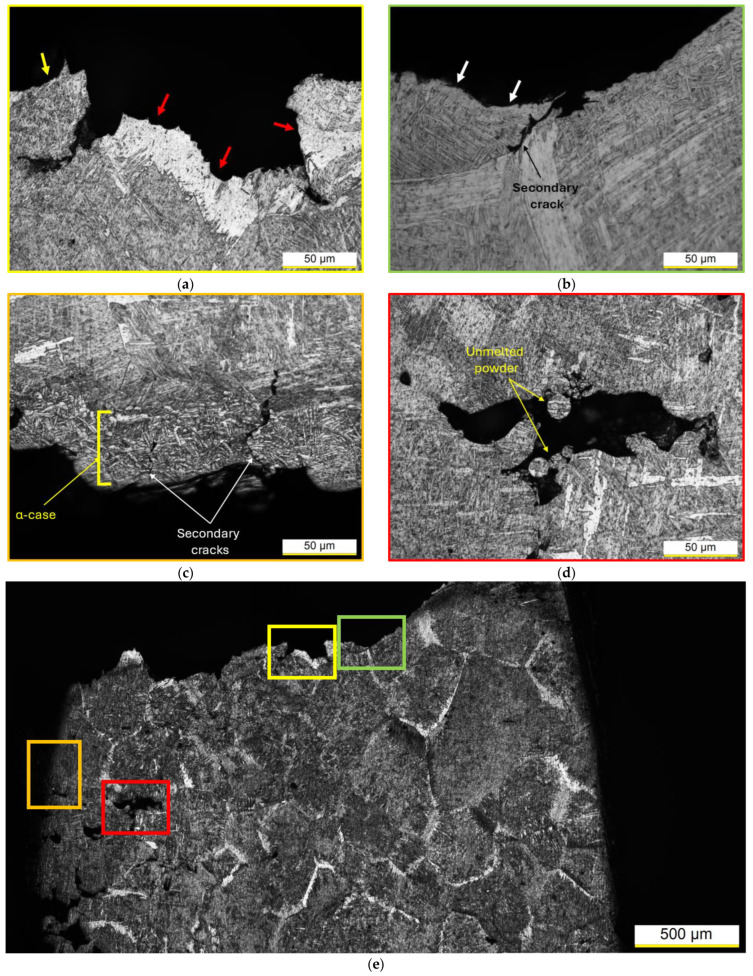
Fracture profile of a Ti64 samples heat-treated at 1050 °C per 60 min. Panels (**a**–**d**) belong to the sectioned tensile sample (**e**) and exhibit (**a**,**b**) fracture profile, (**c**) edge of the sample with α-case layer, and (**d**) lack-of-fusion pore. Yellow, red, and white arrows indicate the crack pathways along the boundaries of the α-phases.

Figure 9a shows the work-hardening capacity of the heat-treated Ti6Al4V samples as a function of their constant strain-hardening exponent. Work-hardening capacity (WHC) is defined as follows:

(10) $$WHC=\frac{\sigma_{UTS}-\sigma_{y}}{\sigma_{y}}=\frac{\sigma_{UTS}}{\sigma_{y}}-1$$

and represents the ability to accommodate dislocations during a plastic deformation. For this reason, the SHTed Ti6Al4V samples show a better capacity to store dislocation compared to those heat-treated below the β-transus temperature due to a greater amount of the bcc β-phase, different grain sizes, and crystal orientations, as supported in [11,31,56,57]. In fact, the fully lamellar structure of the annealed Ti6Al4V samples limits strain-hardening ability and negative effects on uniform elongation [29,58,59]. Conversely, the SHTed Ti6Al4V alloy exhibits a lower degree of elasticity (R obtained from Equation (6) and shown in Figure 9a) and, thus, a higher capacity for plastic deformation before failure compared to the annealed alloy. Figure 9b shows the variation of the softening factor, which represents the capacity to recover dislocation during deformation (Figure 4), in relation to the true plastic strain (Equation (9)) [53,60,61,62,63]. From the plotted results, it can be concluded that all Ti6Al4V samples analyzed in the present work exhibit higher elasticity compared to those produced by electron beam powder bed fusion (square-shaped red symbols, [60,61]), as well as those produced by laser powder bed fusion both in as-built (circle-shaped symbols [53,61,62,63]) and heat-treated (hexagonal-shaped symbols [61,62]) conditions. Specifically, the columnar β-grains that characterize the annealed Ti6Al4V samples conferred similar true elongation strains to the as-built LPBFed Ti6Al4V samples, but the decomposed α′-martensite laths may increase both the softening factor and elasticity. The higher amount of β-phase and the presence of an α-β microstructure, arranged into colonies or Widmanstätten structure, enhance the true plastic strain at the expense of the softening factor. Considering that both the as-built EBM and heat-treated LPBF Ti6Al4V samples exhibit an α + β microstructure within the β-grains, it is possible to conclude that the mechanical behavior of SHTed Ti6Al4V is more influenced by the α + β structure (a,b) and the α-case (Figure 10c) than by the morphology of the β-grains. 

As highlighted in Figure 10, the main crack appears to propagate indeed along the boundaries of both the α + β colonies (red arrows in Figure 10a) and the α + β laths (yellow arrows in Figure 10a), as well as along the boundaries of the coarsened α-phase (white arrows in Figure 10b). In summary, cracks propagate across the softer bcc β-phase, as extensively discussed in [6]. Furthermore, mechanical behavior can be also influenced by the presence of the irregular-shaped lack of fusion (Figure 10d), thoroughly argued in [6]. Lastly, Figure 10e summarizes Figure 10a–d and clearly highlights the cross-sectional area reduction due to the necking instability conditions that occurred during the tensile test. 

**Figure 11:**
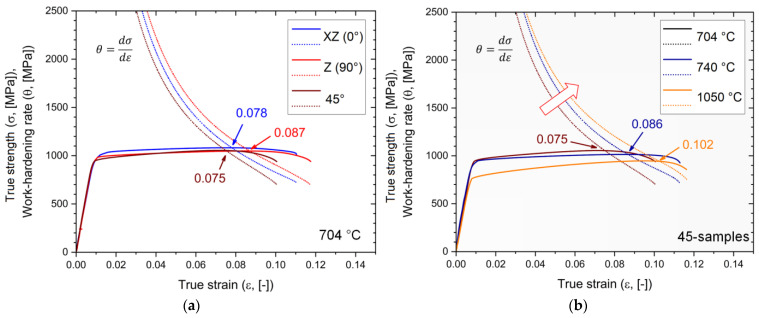
Representative true stress–strain curves and work-hardening rates of (**a**) XZ-, Z-, and 45-samples heat-treated at 704 °C, (**b**) 45-samples heat-treated at 704 °C (black lines), 740 °C (blue lines), and 1050 °C (orange lines).

Considering that the power-law equation (Equation (1)) effectively describes the plastic region (see Figure 3b), the Considère criterion can be applied to evaluate the uniform elongation and the potential neck formation in the Ti6Al4V samples. Each work-hardening curve was obtained by Equation (2), where the strain-hardening exponents are considered as constant value (see linear fit in Figure 3a), ensuring the satisfaction of Equations (2)–(4). Indeed, only work-hardening curves obtained by constant n and K values intersect the true stress–strain curves at the $\left(\sigma_{UTS},\;\varepsilon_{UTS}\right)$ point (Figure 11). Furthermore, these intersections between the work-hardening curve $\left(\theta =-\frac{d\sigma }{d\varepsilon }\right)$ and the true stress–strain curves confirm the onset of the necking instability in all analyzed Ti6Al4V samples, regardless of the post-processing heat treatments (Figure 11). Focusing on the Ti6Al4V samples annealed at 704 °C (Figure 11a), slight variations in uniform elongation values are observed due to the different build orientations. A clear increase in uniform elongation was obtained by increasing the heat-treatment temperatures while maintaining constant build orientation (red arrow in Figure 11b). 

**Figure 12:**
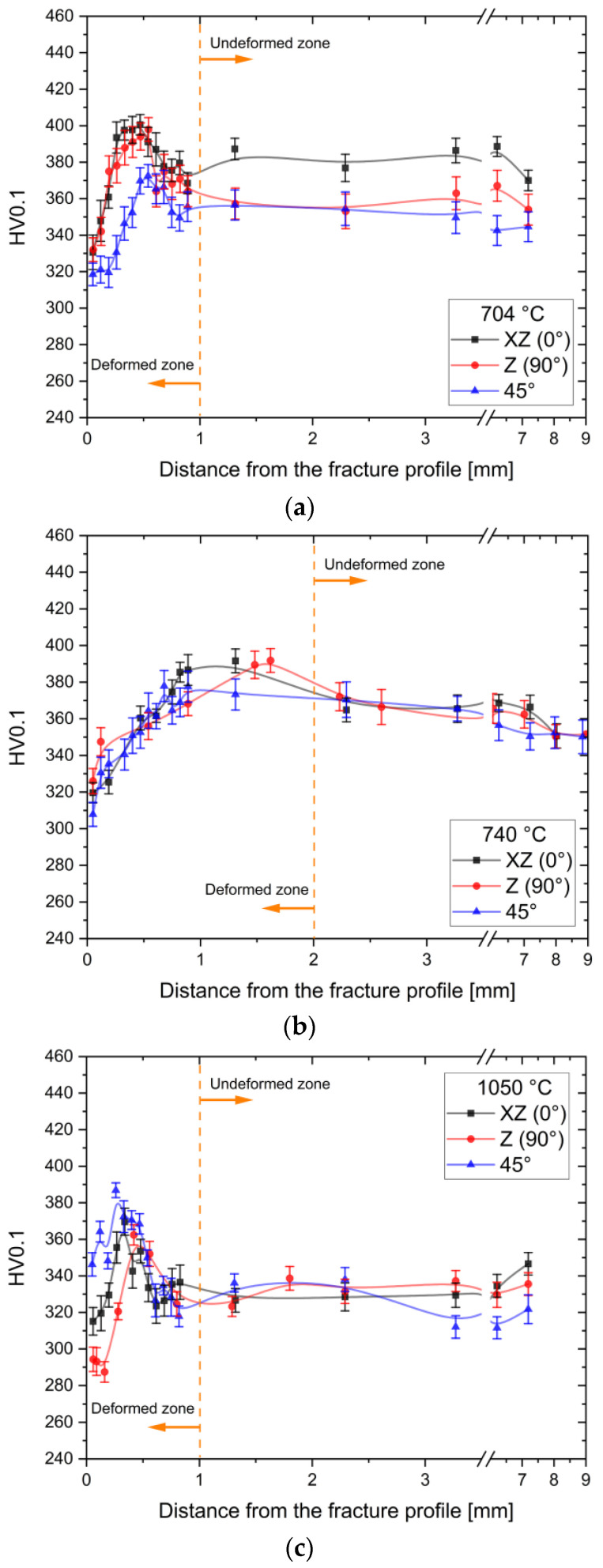
Vickers microhardness profiles (see) measured on the cross-section of the broken dog-bone samples after the heat treatments at 704 °C (**a**), 740 °C (**b**), and 1050 °C (**c**). Orange dotted lines divide the highly deformed zone and the undeformed one.

Figure 12 displays the Vickers microhardness profiles (see) of the tested Ti64 samples after the exposure at 704 °C (Figure 12a), 740 °C (Figure 12b), and 1050 °C (Figure 12c). Starting from the farthest point of each profile, Vickers microhardness trends increase up to a maximum value due to the work-hardening in the necking region. From this point, hardness values decrease up to the closest zone to the fracture profile, probably due to the presence of several damages that are undetectable through optical microscopy. The high strain rates characteristic of the closest zone to the fracture profile can induce the formation of several secondary cracks and tears [27,64,65]. Considering the Vickers microhardness profiles plotted in Figure 12a,b, it appears that Ti6Al4V samples heat-treated at 740 °C exhibit a slightly larger damaged area compared to those at 740 °C (Figure 12a). These zones are located between the fracture profile and the maximum value of the Vickers profile. As discussed in Figure 8 and Figure 9a, the highest work-hardening capacity observed in the SHTed Ti6Al4V samples is also reflected in the greatest variation in hardness between the maximum and the undeformed region (Figure 12c). In this context, it is important to note that the undeformed region may still contain a certain amount of strain that is not detectable through the Vickers profile [64,65]. 

Lastly, the average microhardness values of the undeformed zones (Figure 12) reflect the decline in mechanical properties (see Table 2) from the Ti6AlV4 samples annealed at 704 °C to those solubilized at 1050 °C. 

**Figure 13:**
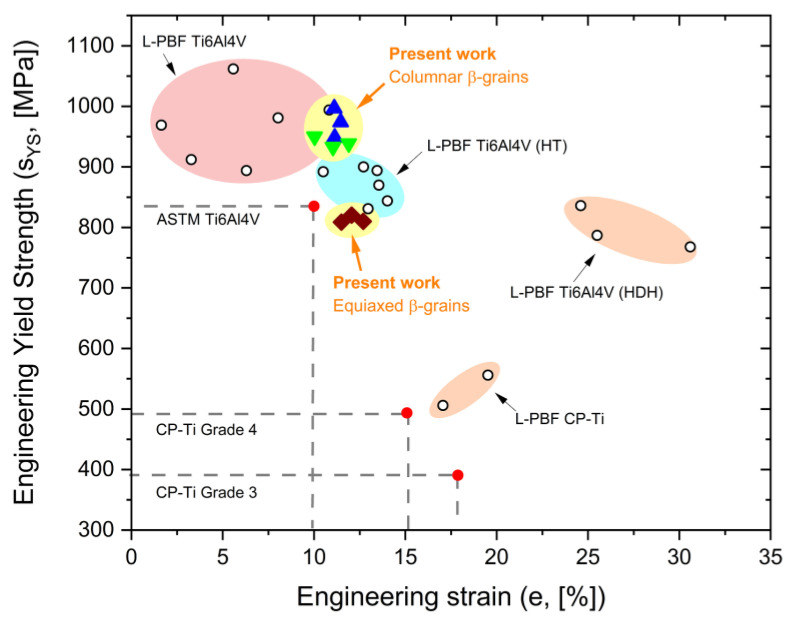
Engineering YS versus engineering strains of the Ti64 samples analyzed in the present work (colored symbols) and compared with Ti64 and CP-Ti (CP: commercially pure) samples studied in [6,66,67,68,69]. HDH-Ti means hydrogenated–dehydrogenated titanium alloy.

Figure 13 correlates the engineering yield strength (s_YS_) to the engineering strain (*e*) of the heat-treated Ti6Al4V samples analyzed in this study (Table 2) and compares these values with the LPBF Ti6Al4V and CP-Ti alloys investigated in [6,66,67,68,69]. It is important to note that the YS of Ti6Al4V samples heat-treated at 704 and 740 °C is comparable to the LPBF Ti6Al4V samples in as-built conditions. Both recrystallization phenomena affecting the columnar β-grains and the argon cooling from the β-region significantly reduce the YS; thereby, the ASMT standard is not satisfied [67]. Therefore, aging heat treatments in the 450–600 °C range can improve mechanical performance by precipitation of the TiAl_3_ and Ti_3_Al phases, as demonstrated in [50]. In contrast, according to the results obtained by using the Considère criterion (Figure 11), all analyzed samples exhibited excellent uniform elongations (>7.8%). These values are higher than those exhibited by Ti6Al4V samples produced by LPBF and powder metallurgy in [7,11,53,70,71], and are comparable to those shown by wrought Ti64 alloys in [11] with the same strain rate range (10^–3^ ÷ 10^–4^ s^–1^). 

## 4. Conclusions

An investigation of the strain-hardening and ductility of laser powder bed-fused Ti6Al4V samples heat-treated below (704 and 740 °C) and above (1050 °C) the β-transus was presented in this paper. The following conclusions can be drawn from the presented results:

1. The microstructure of the annealed Ti6Al4V samples exhibits retained columnar β-grains containing α-phase laths arranged in a cross-hatched structure. When treatment temperature increases from 704 to 740 °C, α-phase width increases by about +30%.
2. The columnar β-grains recrystallize into equiaxed β-grains during the exposure at 1050 °C, and subsequent argon cooling forms an α + β microstructure with several morphologies: (i) colonies, (ii) Widmanstätten structure, and (iii) globular.
3. Due to the rise in heat-treatment temperature, both yield and ultimate tensile strengths decreased by about—16% and—12%, respectively. However, elongation values did not show significant improvement. All analyzed samples exhibited excellent uniform elongations (>7.8%), which increased from Ti64 samples annealed at 704 °C to those solution-heat-treated at 1050 °C, along with necking instability.
4. The equality between the uniform elongations obtained by the Considère criterion and the $\varepsilon_{UTS}$ indicates that constant n and K values must be considered.
5. Annealed samples show higher $\sigma_{YS}/\sigma_{UTS}$ ratios than those exhibited by the Ti6Al4V samples with recrystallized microstructure. The consequent lower work-hardening capacity values promote low work-hardening of the necking region. Thereby, the recrystallized microstructure shows both lower work-hardening rates in Stage II and lower softening values (i.e., dynamic recovery). This is reflected in the highest hardness increment from the undeformed region obtained during the plastic deformation.
6. Despite the anisotropy conferred by the build orientations, they did not significantly influence the work-hardening behavior.

Future works should focus on the work-hardening behavior of aged samples, given that the tensile strength of solution-heat-treated Ti6Al4V samples must be improved.

## Figures and Tables

**Table 1:** Chemical composition (wt.%) of the gas-atomized Ti64 powder.

Ti	Al	V	Fe	C	N	O
Bal.	6.5	4.1	0.21	0.01	0.01	0.1

**Table 2:** Average of engineering UTS (s_UTS_), YS (s_YS_), and elongation (e (%)) values of the heat-treated XZ-, Z-, and 45-samples.

Samples	s_UTS_ (MPa)			s_YS_ (MPa)			e (%)		
	704 °C	740 °C	1050 °C	704 °C	740 °C	1050 °C	704 °C	740 °C	1050 °C
XZ	1059 ± 2	1008 ± 4	909 ± 6	997 ± 21	950 ± 14	820 ± 12	11 ± 2	10 ± 1	12 ± 1
Z	1043 ± 14	1000 ± 8	924 ± 3	974 ± 9	939 ± 10	809 ± 6	11 ± 1	12 ± 1	12 ± 1
45°	1022 ± 10	1002 ± 12	930 ± 11	949 ± 22	933 ± 6	810 ± 11	11 ± 1	11 ± 1	13 ± 1

**Table 3:** Maximum values of work-hardening rate ($\theta_{max}$ × 10^3^ (MPa)) obtained by heat-treated Ti64 samples.

	704 °C	740 °C	1050 °C
XZ	3.7 ± 0.2	3.8 ± 0.2	2.5 ± 0.2
Z	3.8 ± 0.1	3.7 ± 0.2	2.6 ± 0.2
45°	3.6 ± 0.2	3.8 ± 0.2	2.5 ± 0.2

## Data Availability

The original contributions presented in the study are included in the article, further inquiries can be directed to the corresponding author.
